# Relationship between dietary protein, serum albumin, and mortality in asthmatic populations: a cohort study

**DOI:** 10.3389/fimmu.2024.1396740

**Published:** 2024-07-04

**Authors:** Rongjuan Zhuang, Jiaxin Liao, Mohan Giri, Jun Wen, Shuliang Guo

**Affiliations:** Department of Respiratory and Critical Care Medicine, The First Affiliated Hospital of Chongqing Medical University, Chongqing Medical University, Chongqing, China

**Keywords:** protein intake, albumin, asthma, mortality, National Health and Nutrition Examination Survey (NHANES)

## Abstract

**Background:**

Currently, there is limited research on the correlation between protein levels in the body and asthma. We used data from the NHANES to explore the relationship of dietary protein, serum albumin, with mortality in individuals with asthma to better understand their impact on asthma.

**Method:**

This investigation involved 3005 individuals with asthma from the NHANES dataset. Studying potential links between dietary protein, serum albumin, and mortality in asthmatic populations utilized the Cox proportional hazards models, trend test, restricted cubic splines (RCS), and Kaplan-Meier survival analysis. Furthermore, subgroup analyses were carried out to explore these connections within specific populations.

**Result:**

After considering all potential variables, multivariate Cox proportional hazard models proved that dietary protein intake did not have an independent connection with all-cause mortality, but serum albumin was inversely linked with all-cause mortality. Each unit rise in serum albumin (g/l) was linked to a 13% decrease in the likelihood of all-cause mortality. RCS confirmed a negative and linear connection of serum albumin with all-cause mortality. The Kaplan-Meier survival curve suggested that asthmatic adults with greater serum albumin levels had a decreased risk of mortality compared to those with lower levels.

**Conclusion:**

The investigation proved a negative linear connection of serum albumin with all-cause mortality in asthma patients. However, there was no independent link discovered between dietary protein intake with mortality. This indicates that serum albumin could be a significant factor in predicting long-term outcomes for asthma patients.

## 1 Introduction

One of the most prevalent chronic non-communicable illnesses worldwide, asthma is characterized by a variety of sporadic and variable symptoms related to bronchospasm and airway inflammation, including dyspnea, tightness in the chest, wheezing, coughing, and sputum (1). Over the past few decades, there has been a significant increase in the prevalence, morbidity, death, and economic burden of asthma worldwide, particularly in children. Globally, asthma claims the lives of about 180,000 individuals each year (2, 3). Some patients develop a partial or whole resistance to glucocorticoids during treatment, necessitating the use of comparatively large doses of glucocorticoids to control their symptoms. In other individuals, increasing loss of lung function is a contributing factor to death (4). More than 1% of all disability-adjusted life years lost globally are estimated to be attributable to asthma (5, 6).

In many industrialized nations, obesity has become an epidemic due to the widespread adoption of the Western diet. Saturated fats, refined carbs, and sodium intake rise when processed meals are consumed frequently and fruits, vegetables, and whole grains are consumed less frequently. Asthma is one of the chronic inflammatory disorders that are more likely to develop in those who follow this kind of poor diet (7). According to certain research, a high intake of protein and fat and a high intake of carbohydrates are inversely connected with lung function (8). For the majority of patients with chronic illnesses, inadequate food consumption that falls short of their needed calorie and protein intake leads to poor quality of life (9).

Albumin has a molecular weight of 66.3 kD (10, 11). Protein A, which is the most prevalent in plasma, is of critical importance in facilitating the movement of charged and electrically neutral molecules and ions and maintaining the colloid osmotic pressure of the blood (12). Vascular permeability increases are a common feature of inflammation. These increases make it easier for large amounts of albumin to pass through the interstitial and epithelial surfaces of many organs, including the lungs (13, 14). Significant elevations in tissue albumin levels might potentially confer advantageous consequences, including the provision of antioxidant defense for tissues (15). Human serum albumin is unique because it has fourteenteen disulfide bonds made up of thirty-four cysteine residues and a free thiol at position Cys-34 (11). This is the main pathway by which albumin scavenges reactive oxygen and nitrogen species (ROS and RNS) and thus participates in redox processes (12). Albumin is regarded as a significant extracellular antioxidant in plasma within this particular context.

Currently, there is insufficient study on the link between protein levels in the body with asthma. So we used NHANES data to investigate the relationship of dietary protein intake, serum albumin levels, and mortality in persons with asthma to better understand their impact on asthma.

## 2 Materials and methods

### 2.1 Study data and population

**Figure 1:**
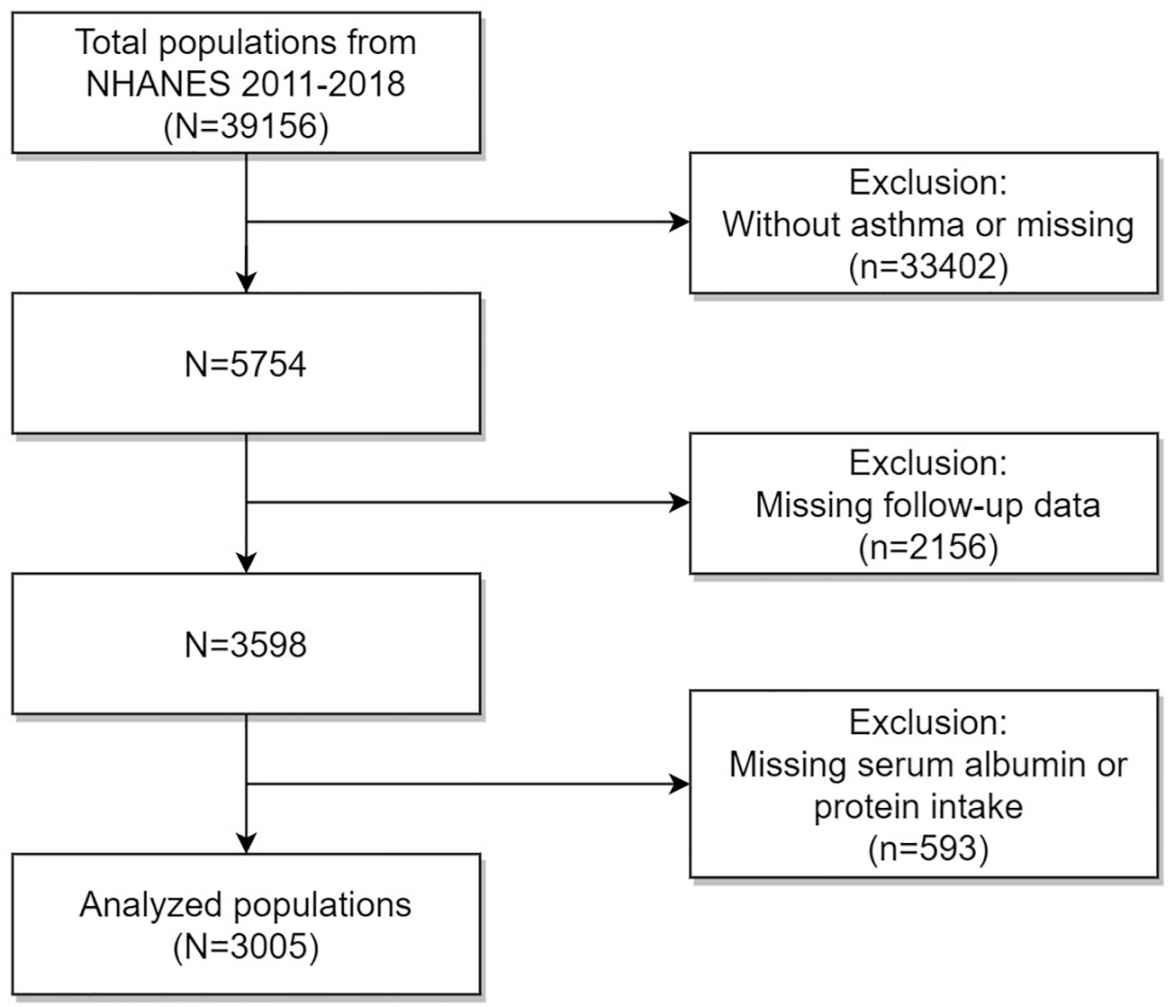
Flow diagram of selecting populations for analysis.

The data for that investigation were collected from the National Health and Nutrition Examination Survey (NHANES) public database, which was managed by the Centers for Disease Control and Prevention (CDC) in the USA. The NHANES database collected vital and health statistics for the country. The NHANES study was conducted using a complex multistage stratified sample design to identify a diverse sample of the US population that was not in institutions. Every participant underwent informed consent prior to the data collection procedures and thorough health examinations. The NHANES study protocol received approval from the National Center for Health Statistics (NCHS)’ Research Ethics Review Board. **Figure 1** illustrates that NHANES had a participation of 39156 people from 2011 to 2018. Adhering to specific inclusion and exclusion criteria, our study population excluded: (1) individuals without asthma or with missing data (n = 33402); (4) those with missing follow-up data (n = 2156); (3) those lacking serum albumin or dietary protein intake (n = 593). Finally, our investigation involved a sample of 3005 asthmatic people in the USA.

### 2.2 All variables

According to the NHANES introduction, participants’ dietary protein and other nutrient intake were evaluated through a 24-hour dietary recall method. A professional technician inquired about the types and quantities of foods and medications consumed within a day, which were then documented in the NHANES computer-assisted dietary survey system. Estimates for the intake of each food component were calculated using the University of Texas Food Intake Analysis System and the USDA Survey Nutrient Database. The DcX800 method, a bichromatic digital endpoint technique, is utilized to evaluate serum albumin concentration. Bromcresol purple (BCP) reagent and albumin mix to create a complex during the process. The system maintains records of any variations in absorbance at 600 nm. The amount of albumin in the sample is directly proportional to the change in absorbance. The death index (NDI), as of December 31, 2018, was used to determine the follow-up status of our study populations. The NCHS offered further details regarding the matching approach. We utilized the 10th edition of the International Statistical Classification of Diseases (ICD-10) to establish mortality status. Our main outcome variable was all-cause mortality.

To mitigate the potential influence of various factors, we included numerous covariates in our study. The covariates considered in the analysis were sex (male and female), age, race, education, poverty to income ratios (PIR), marriage, body mass index (BMI), smoking status, intake of alcohol, energy, total sugar and total fat, hypertension history, diabetes history, cardiovascular disease (CVD) history, chronic obstructive pulmonary disease (COPD) history, cancer history, glucocorticoid drug (whether glucocorticoid drugs were used in the past month), serum creatinine, white blood cell number (WBC), blood neutrophils number (BNEU), and blood eosinophils number (BEOS), and serum total protein. Has a doctor or other healthcare provider ever diagnosed you with asthma? was one of the questions on standardized questionnaires given to participants during their visits. Participants who answered positively were classified as having a diagnosis of asthma. The NHANES website provides a comprehensive description of laboratory procedures.

### 2.3 Statistical analysis

Statistical analyses were performed utilizing R software. P < 0.05 was regarded as statistical significance. Initially, we built three univariate and multivariate Cox proportional hazards regression models to assess the independent association between dietary protein, serum albumin, and all-cause mortality in individuals with asthma. Three constructed models were as follows: Model X (adjusted for none), Model Y (adjusted for age, race, sex, education, marriage, and PIR), and Model Z (adjusted for sex, age, race, education, marriage, PIR, BMI, smoking, intake of alcohol, energy, total sugar and total fat, hypertension history, diabetes history, CVD history, COPD history, cancer history, glucocorticoid drug, serum creatinine, WBC, BNEU, BEOS, and serum total protein). Subsequently, we utilized the trend test and restricted cubic spline (RCS) to examine whether the correlation was linear. Meanwhile, stratified analyses were carried out to explore the connection between serum albumin and all-cause mortality across diverse asthmatic populations. Lastly, we also carried out the Kaplan-Meier analysis to explore the prognostic impact of serum albumin on all-cause mortality in asthmatics. Multiple imputation was used to handle variables with missing values, and the total proportion of missing values for each covariate was less than 10%.

## 3 Results

### 3.1 Baseline characteristics

The investigation involved 3005 individuals with asthma (1269 men and 1736 women), and their characteristics were analyzed based on follow-up outcomes (**Table 1**). Our investigation found that the average age of participants was 45.66 years old, with the majority of the population being white populations. The average period of follow-up for populations was 57.96 months. Variations in the distributions of age, race, education, marriage, PIR, smoking, intake of energy and fat, hypertension, diabetes, CVD, COPD, cancer, glucocorticoid drugs, serum creatinine, WBC, BNEU, serum albumin, and serum total protein were found to be statistically significant among the follow-up outcome groups (**Table 1**). However, there were no significant variations in sex, BMI, alcohol consumption, sugar intake, protein intake, or BEOS (p > 0.05). In comparison to the survival populations, the death populations exhibited reduced serum albumin levels.

**Table 1:** Baseline characteristics based on survival status.

	Survival	Death	P value
Sex (%)			0.073
Male	1186 (41.83%)	83 (48.82%)	
Female	1649 (58.17%)	87 (51.18%)	
Age (years)	44.46 ± 18.03	65.67 ± 15.53	<0.001
Race (%)			<0.001
Other race populations	948 (33.44%)	34 (20.00%)	
White populations	1161 (40.95%)	95 (55.88%)	
Black populations	726 (25.61%)	41 (24.12%)	
Education (%)			0.002
Less than high school	552 (19.47%)	51 (30.00%)	
High school	636 (22.43%)	40 (23.53%)	
More than high school	1647 (58.10%)	79 (46.47%)	
Marriage (%)			0.008
Married	1336 (47.13%)	62 (36.47%)	
Single	1271 (44.83%)	97 (57.06%)	
Living with a partner	228 (8.04%)	11 (6.47%)	
PIR	2.36 ± 1.84	1.89 ± 1.76	0.001
BMI (kg/m2)	30.82 ± 8.30	30.80 ± 8.95	0.979
Smoking status (%)			<0.001
Smoker	1257 (44.34%)	105 (61.76%)	
Non-smoker	1578 (55.66%)	65 (38.24%)	
Alcohol intake (gm)	9.94 ± 29.33	8.48 ± 26.48	0.524
Energy intake (kcal)	2146.42 ± 1072.36	1945.25 ± 1025.30	0.017
Sugar intake (gm)	114.35 ± 83.90	111.98 ± 90.41	0.722
Fat intake (gm)	84.09 ± 50.54	74.06 ± 43.37	0.011
Protein intake (gm)	80.87 ± 45.93	73.90 ± 48.12	0.055
Fat intake (gm)	78.06 ± 53.27	66.62 ± 46.78	0.004
Hypertension (%)			<0.001
No	1755 (61.90%)	43 (25.29%)	
Yes	1080 (38.10%)	127 (74.71%)	
Diabetes (%)			<0.001
No	2429 (85.68%)	110 (64.71%)	
Yes	406 (14.32%)	60 (35.29%)	
CVD history (%)			<0.001
No	2458 (86.70%)	95 (55.88%)	
Yes	377 (13.30%)	75 (44.12%)	
COPD history (%)			<0.001
No	2577 (90.90%)	115 (67.65%)	
Yes	258 (9.10%)	55 (32.35%)	
Cancer history (%)			<0.001
No	2562 (90.37%)	126 (74.12%)	
Yes	273 (9.63%)	44 (25.88%)	
Glucocorticoid drugs (%)			<0.001
No	2405 (84.83%)	108 (63.53%)	
Yes	430 (15.17%)	62 (36.47%)	
Serum creatinine (umol/l)	77.63 ± 33.29	104.39 ± 120.64	<0.001
WBC (1000 cells/uL)	7.47 ± 2.38	8.10 ± 3.76	0.001
BNEU (1000 cell/uL)	4.39 ± 1.83	5.09 ± 2.18	<0.001
BEOS (1000 cells/uL)	0.23 ± 0.17	0.25 ± 0.23	0.092
Serum albumin (g/L)	42.02 ± 3.66	39.97 ± 4.18	<0.001
Serum total protein (g/L)	71.17 ± 4.62	69.80 ± 6.17	<0.001

Continuous and categorical variables were displayed individually as mean ± SD or proportions. HR, hazard ratio; CI, confidence interval; PIR, poverty to income ratios; CVD, cardiovascular disease; COPD, chronicobstructive pulmonary disease; WBC, white blood cell; BNEU, blood neutrophils; BEOS, blood eosinophils.

### 3.2 Association between dietary protein, serum albumin, and all-cause mortality

We applied univariate and multivariate Cox proportional hazard models to investigate the correlation of dietary protein intake, serum albumin, and all-cause mortality in asthmatics (**Table 2**). In the univariate Cox hazard model (Model X), without adjustment of covariates, dietary protein intake was inversely related to all-cause mortality. But in the multivariate Cox hazard models (Model Y and Z), with the adjustment of diverse covariates, dietary protein intake was not independently related to all-cause mortality. It was found that serum albumin was negatively linked to all-cause mortality in both univariate and multivariate Cox hazard models (Models X, Y, and Z), even after different covariates were taken into account. In Model Z, which controlled all covariates, the risk of all-cause death decreased by 13% for every additional unit of serum albumin (g/L). Also, the trend test of the correlation between serum albumin and all-cause mortality was statistically significant in all three models (p for trend < 0.05), which meant that serum albumin was linked to all-cause mortality in asthmatics in a way that was both linear and inverse.

**Table 2:** Association between dietary protein intake, serum albumin, and all-cause mortality in asthmatics.

	Model X	Model Y	Model Z
	HR (95% CI) P value	HR (95% CI) P value	HR (95% CI) P value
Protein intake	1.00 (0.99, 1.00) 0.0257	1.00 (1.00, 1.00) 0.8251	1.00 (1.00, 1.01) 0.1972
Protein intake quartile groups			
Q1	Reference	Reference	Reference
Q2	0.83 (0.56, 1.23) 0.3461	0.81 (0.54, 1.20) 0.2910	0.83 (0.54, 1.28) 0.4094
Q3	0.59 (0.39, 0.91) 0.0161	0.74 (0.47, 1.15) 0.1815	0.76 (0.45, 1.27) 0.2926
Q4	0.60 (0.39, 0.91) 0.0171	0.94 (0.59, 1.48) 0.7804	1.09 (0.56, 2.13) 0.7983
P for trend	0.0055	0.6017	0.8611
Serum albumin	0.84 (0.81, 0.87) <0.0001	0.86 (0.82, 0.90) <0.0001	0.87 (0.83, 0.92) <0.0001
Serum albumin quartile groups			
Q1	Reference	Reference	Reference
Q2	0.66 (0.45, 0.97) 0.0340	0.73 (0.49, 1.08) 0.1122	0.75 (0.50, 1.13) 0.1716
Q3	0.39 (0.25, 0.60) <0.0001	0.47 (0.30, 0.73) 0.0008	0.51 (0.32, 0.81) 0.0042
Q4	0.23 (0.15, 0.36) <0.0001	0.36 (0.23, 0.57) <0.0001	0.41 (0.25, 0.69) 0.0007
P for trend	<0.0001	<0.0001	0.0002

Model X adjusted for none. Model Y adjusted for age, race, sex, education, marriage, and PIR. Model Z adjusted for all covariates. HR, hazard ratio; CI, confidence interval.

### 3.3 Dose-response relationship

**Figure 2:**
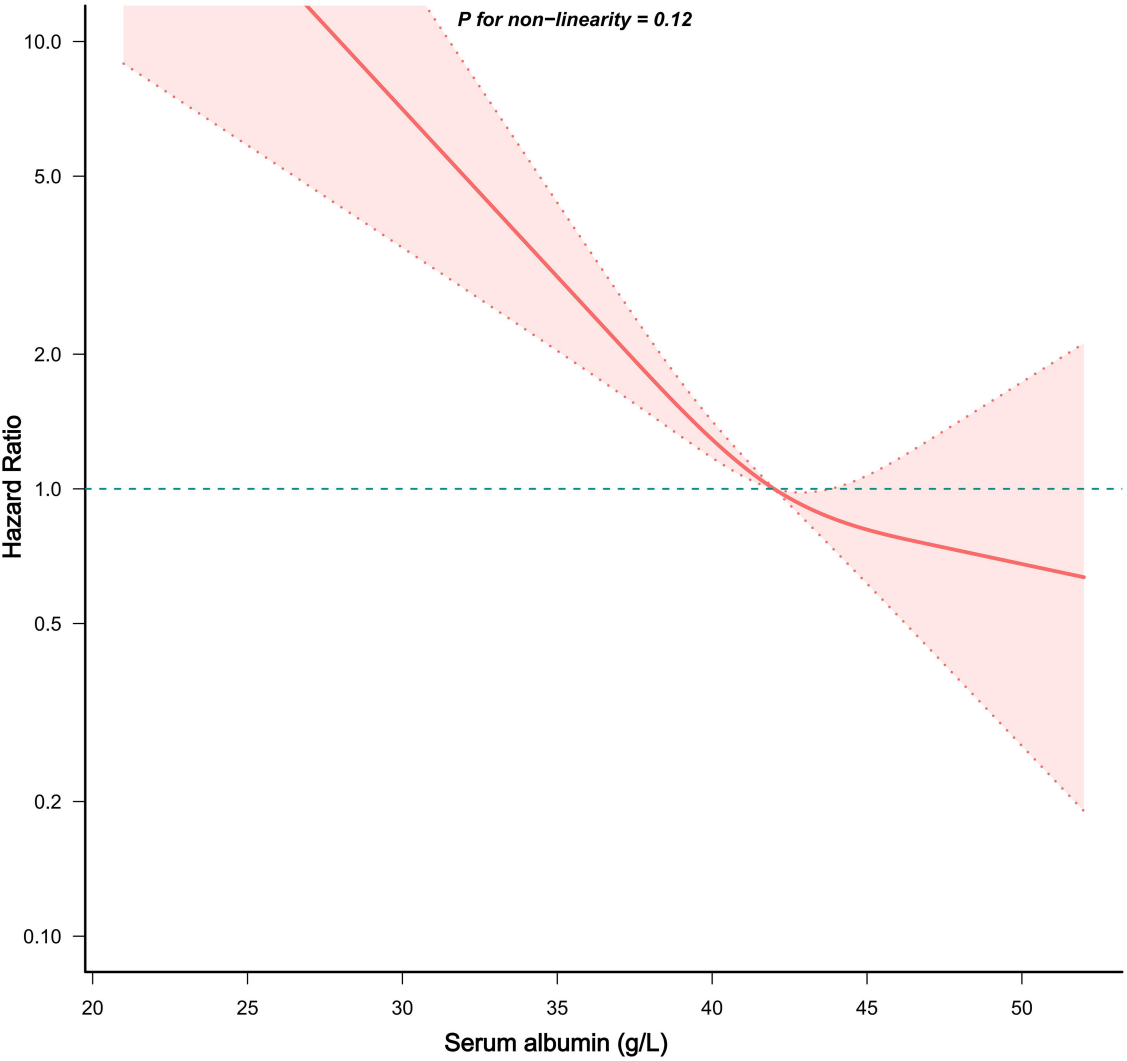
Dose-response correlation between serum albumin and mortality in asthmatics. The red solid line and red area represented the HRs and their accompanying 95%CIs, respectively.

The restricted cubic spline (RCS) was especially sensitive to identifying linear or nonlinear correlation. Hence, we used RCS based on Model Z to ascertain whether the connection between serum albumin and all-cause mortality in asthmatics was linear or not (**Figure 2**). After adjustment for all covariates, we observed a linear and negative correlation between serum albumin and all-cause mortality in asthmatics (P for non-linearity > 0.05).

### 3.4 Subgroup analyses

Subgroup analyses were carried out to evaluate the correlation between serum albumin and mortality throughout various asthmatic populations. The outcomes, grouped by sex, age, race, history of hypertension, diabetes, CVD, COPD, cancer history, and usage of glucocorticoids, were displayed in **Table 3**. A linear and inverse correlation was observed among all asthmatic subgroup populations except for white populations age<40, with a BMI of 25–30, and with a cancer history. And serum albumin and cancer history might have interaction effects with all-cause mortality (p for interaction < 0.05).

**Table 3:** Stratified associations between serum albumin and all-cause mortality in asthmatics.

Subgroup	N	HR (95% CI) P value	P for interaction
**Sex**			0.202
Male	1269	0.83 (0.77~0.89) <0.001	
Female	1736	0.89 (0.83~0.95) <0.001	
**Age**			0.541
<40	1274	1.01 (0.83~1.22) 0.959	
40-60	873	0.87 (0.78~0.97) 0.012	
≥60	858	0.85 (0.8~0.9) 0.001	
**Race**			0.093
White populations	1256	0.95 (0.88~1.03) 0.201	
Black populations	767	0.87 (0.79~0.95) 0.002	
Other race populations	982	0.77 (0.7~0.86) 0.001	
**BMI**			0. 843
<25	766	0.84 (0.76~0.92) <0.001	
25-30	823	0.94 (0.84~1.04) 0.234	
≥30	1416	0.87 (0.8~0.93) <0.001	
**Hypertension**			0.134
No	1798	0.84 (0.76~0.93) 0.001	
Yes	1207	0.88 (0.83~0.93) 0.001	
**Diabetes**			0.825
No	2539	0.88 (0.83~0.94) <0.001	
Yes	466	0.84 (0.76~0.92) <0.001	
**CVD history**			0.082
No	2553	0.91 (0.85~0.97) 0.006	
Yes	452	0.83 (0.77~0.89) <0.001	
**COPD history**			0.146
No	2692	0.85 (0.8~0.9) <0.001	
Yes	313	0.9 (0.83~0.99) 0.023	
**Cancer history**			0.001
No	2688	0.82 (0.78~0.87) <0.001	
Yes	317	1.05 (0.94~1.17) 0.416	
**Glucocorticoid drugs**			0.382
No	2513	0.85 (0.8~0.9) 0.001	
Yes	492	0.89 (0.81~0.97) 0.009	

All stratified analyses adjusted for all covariates except for the stratification variable. HR, hazard ratio; CI, confidence interval; CVD, cardiovascular disease; COPD, chronic obstructive pulmonary disease.

### 3.5 Kaplan–Meier survival curve

**Figure 3:**
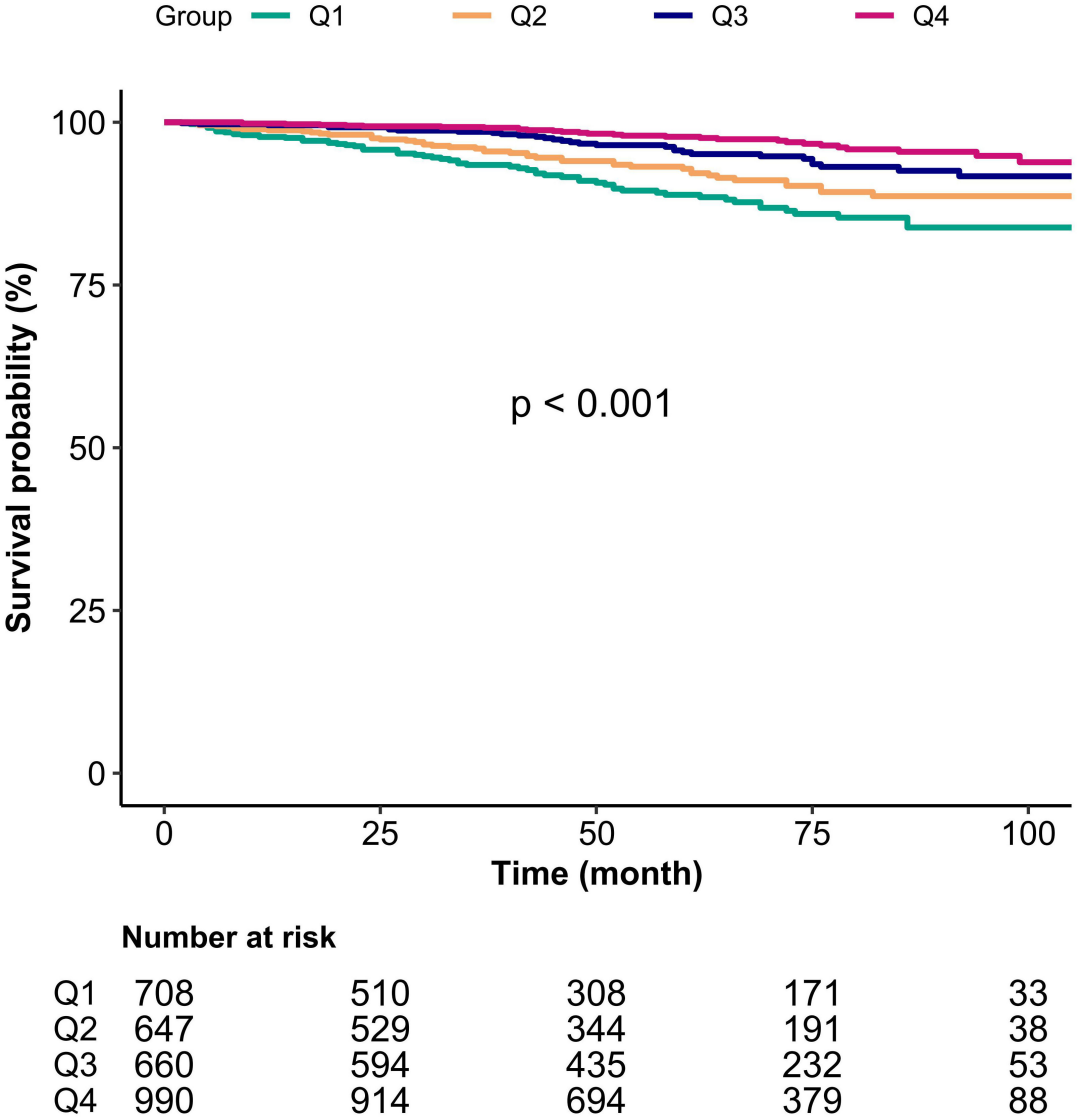
Kaplan-Meier analysis of mortality by groups of serum albumin quartile in asthmatics. Q1-Q4, Grouped by serum albumin quartiles.

In **Figure 3**, we carried out a Kaplan-Meier analysis based on serum albumin quartile groups to examine the prognostic impact of serum albumin on mortality in asthmatics. We observed that the risk of mortality was lower for asthmatic adults with the highest serum albumin quartiles group (Q4), compared with asthmatics with the lowest serum albumin quartiles group (Q1).

## 4 Discussion

In this investigation, we selected 3005 NHANES participants with asthma based on designed exclusion criteria. In the follow-up outcome group, the difference in the distribution of serum albumin was statistically significant, whereas there was no significant difference in protein intake. Serum albumin levels were reduced in the death population compared with the survival population. We used univariate and multivariate Cox proportional hazard models to investigate the connection between dietary protein intake, serum albumin and all-cause mortality in patients with asthma. The results suggested that dietary protein intake was not independently related to all-cause mortality, whereas serum albumin was negatively related to all-cause mortality (P < 0.05). In model Z, which controlled for all covariates, each unit increase in serum albumin (g/l) was associated with a 13% reduction in the risk of all-cause mortality. RCS confirmed a linear negative connection of serum albumin with all-cause mortality in asthmatics. The results of subgroup analyses suggested a linear negative connection of serum albumin with all-cause mortality in all subgroups, except for white populations age<40, with a BMI of 25–30, and with a cancer history. And there was no interaction among all subgroups. Kaplan-Meier survival curves suggested asthmatic populations in the higher serum albumin quartile (Q4) had a lower risk of death compared with asthmatics in the lower groups (Q1).

In this study group, higher age, lower income, smoking, and co-morbidities were associated with a higher risk of death in asthma patients. This is consistent with the findings of many previous studies (16, 17). Several previous studies have found that lower albumin levels are associated with increased mortality in both the short and long term (18, 19). The negative association of serum albumin levels with mortality in long-term follow-up has also been found in elderly people (20, 21), post-surgical patients (19), patients after acute myocardial infarction (22), and patients with chronic kidney disease (23), which is consistent with our findings. However, our study considered a wider variety of potential factors, applied a more rigorous statistical approach and confirmed a linear association between serum albumin and mortality in asthmatics. In addition, we utilized stratified analyses to investigate the connection of serum albumin levels and all-cause mortality in various groups to avoid the impact of confounding variables.

Serum albumin levels are reduced in various conditions, such as inflammation, protein malnutrition, end-stage liver disease, or malignancy (24). Human serum albumin (HSA) is a cysteine-rich serum protein that is taken up by many cells through receptor-mediated liquid-phase endocytosis. This biological process has been documented in lung epithelial cells and may occur in other cell types (25). It has been demonstrated that albumin can protect cells from oxidative damage and regulate nuclear factor kappa-B (NF-κB) activation by specifically regulating cellular glutathione (GSH) levels (26). In previous studies, abnormal CXCR4/CXCL12 signaling was involved in a variety of pathophysiological processes, such as cancer and organismal inflammation. The natural fragment of serum albumin, EPI-X4, has been identified as an endogenous peptide antagonist and inverse agonist of CXCR4, and novel CXCR4 antagonists developed on the basis of this feature can be used in the treatment of atopic dermatitis, asthma, and other CXCR4-related diseases (27).

Serum albumin is extensively involved in the development of various respiratory diseases. Capillary bronchiolitis is the leading cause of infant hospitalization in the USA and is accompanied by life-threatening apnea. It has been suggested that low serum albumin levels appear to be associated with an increased risk of apnea and are potentially a predictor of future apnea (28). A cross-sectional study that included 3286 individuals found a positive correlation between serum albumin levels and lung function, including forced vital capacity (FVC) and forced expiratory volume in one second (FEV 1) (29). Decreased blood albumin levels are a well-known clinical indication of malnutrition, resulting in weakened respiratory muscles and diminished lung function (29, 30). Moreover, chronic obstructive pulmonary disease (COPD) is a progressive disease characterized by chronic respiratory inflammation and parenchymal lung damage. Biological processes such as systemic inflammation and oxidative stress are involved in the pathogenesis of COPD. Serum albumin is a negative acute-phase protein with antioxidant properties. A systematic review and meta-analysis revealed that serum albumin concentrations were significantly depressed in patients with stable COPD compared to non-COPD controls. This result suggests that systemic anti-inflammatory and antioxidant defense mechanisms are defective in patients with COPD (31). A study on anti-oxidative stress in obstructive sleep apnea (OSA) patients found that their serum albumin levels were lower. This may make oxidative stress worse, which can lead to heart and metabolic diseases (32). Besides that, serum albumin is an independent predictor of the severity of lung necrosis in children with community-acquired necrotizing pneumonia (NP) (33).

Our investigation uniquely concentrated on exploring the connection of dietary proteins, serum albumin, and mortality in asthmatics, a topic that has not been previously addressed. Our study includes a substantial number of asthma patients that are nationally representative and considers a wide variety of potential factors that could affect the results. We showed a reverse linear connection of blood albumin levels and overall mortality in individuals with asthma using multivariate Cox hazard models and RCS. Our work provides fresh insights for future research on managing and treating asthma.

Still, we recognize that there are some constraints to our study. Our study has a nationwide sample size; however, most of the data was gathered from the population of the USA. Varying levels of economic development in countries result in diverse food patterns that significantly influence people’s nutritional status and disease rates. Due to the limitations of the NHANES database, we included asthmatic individuals based on questionnaire data as opposed to the lung function test. The 24-hour dietary recall approach records food intake over a 24-hour period, which might not accurately reflect patients’ regular eating patterns and may be subject to memory bias. Serum albumin levels are reduced in various conditions, thus it is a non-specific and prognostically relevant marker of asthma. Cohort studies can establish a causal association between dietary protein, serum albumin, and mortality in asthma patients. However, there are unmeasured and unknown factors that may influence the study results.

## 5 Conclusion

This investigation proved a negative linear connection of serum albumin with all-cause mortality in asthma patients. However, there was no independent link discovered between dietary protein intake and mortality. This indicates that serum albumin could be a significant factor in predicting long-term outcomes for asthma patients.

## Data availability statement

Publicly available datasets were analyzed in this study. This data can be found here: All data can be accessed via the NHANES official website (http://www.cdc.gov/nchs/nhanes/index.htm).

## Ethics statement

The studies involving humans were approved by Prior to implementing the data collection methodologies and conducting comprehensive health assessments, all participants willingly completed informed consent. The NHANES study protocol received approval from the Research Ethics Review Board of the NCHS (Ethical approval number: Protocol #2011-17, Protocol #2018-01). The studies were conducted in accordance with the local legislation and institutional requirements. The participants provided their written informed consent to participate in this study.

## Author contributions

RZ: Writing – original draft, Methodology, Investigation, Conceptualization. JL: Writing – original draft, Software, Methodology, Investigation. MG: Writing – review & editing, Software, Methodology, Data curation. JW: Writing – original draft, Software, Methodology, Formal analysis, Data curation, Conceptualization. SG: Writing – review & editing, Project administration, Methodology, Funding acquisition, Conceptualization.
